# Combined Profiling of Transcriptome and DNA Methylome Reveal Genes Involved in Accumulation of Soluble Sugars and Organic Acid in Apple Fruits

**DOI:** 10.3390/foods10092198

**Published:** 2021-09-16

**Authors:** Wenfang Ma, Baiyun Li, Litong Zheng, Yunjing Peng, Rui Tian, Yangyang Yuan, Lingcheng Zhu, Jing Su, Fengwang Ma, Mingjun Li, Baiquan Ma

**Affiliations:** State Key Laboratory of Crop Stress Biology for Arid Areas/Shaanxi Key Laboratory of Apple, College of Horticulture, Northwest A&F University, Yangling 712100, Shaanxi, China; mawenfang@nwafu.edu.cn (W.M.); libaiyun@nwsuaf.edu.cn (B.L.); zhenglitong@nwafu.edu.cn (L.Z.); yunjingpeng@nwafu.edu.cn (Y.P.); TianRui@nwafu.edu.cn (R.T.); yy.yuan@nwsuaf.edu.cn (Y.Y.); zhulingcheng316@nwsuaf.edu.cn (L.Z.); sujing@nwsuaf.edu.cn (J.S.); fwm64@nwsuaf.edu.cn (F.M.); limingjun@nwsuaf.edu.cn (M.L.)

**Keywords:** *Malus domestica*, DNA methylation, transcriptomics, transcript profiling, fruit acidity, soluble sugar content

## Abstract

Organic acids and soluble sugars are the major determinants of fruit organoleptic quality. Additionally, DNA methylation has crucial regulatory effects on various processes. However, the epigenetic modifications in the regulation of organic acid and soluble sugar accumulation in apple fruits remain uncharacterized. In this study, DNA methylation and the transcriptome were compared between ‘Honeycrisp’ and ‘Qinguan’ mature fruits, which differ significantly regarding soluble sugar and organic acid contents. In both ‘Honeycrisp’ and ‘Qinguan’ mature fruits, the CG context had the highest level of DNA methylation, and then CHG and CHH contexts. The number and distribution of differentially methylated regions (DMRs) varied among genic regions and transposable elements. The DNA methylation levels in all three contexts in the DMRs were significantly higher in ‘Honeycrisp’ mature fruits than in ‘Qinguan’ mature fruits. A combined methylation and transcriptome analysis revealed a negative correlation between methylation levels and gene expression in DMRs in promoters and gene bodies in the CG and CHG contexts and in gene bodies in the CHH context. Two candidate genes (*MdTSTa* and *MdMa11*), which encode tonoplast-localized proteins, potentially associated with fruit soluble sugar contents and acidity were identified based on expression and DNA methylation levels. Overexpression of *MdTSTa* in tomato increased the fruit soluble sugar content. Moreover, transient expression of *MdMa11* in tobacco leaves significantly decreased the pH value. Our results reflect the diversity in epigenetic modifications influencing gene expression and will facilitate further elucidating the complex mechanism underlying fruit soluble sugar and organic acid accumulation.

## 1. Introduction

Fruit taste is influenced by the content of organic acids, soluble sugars, and volatile compounds, which also affects the overall fruit organoleptic quality [1,2]. The concentration of soluble sugars and organic acids in ripening fruits depend on the balance between biosynthesis, degradation, and vacuolar storage. Furthermore, different sugars, such as sucrose, fructose, and glucose, impart significantly different sweetness [3,4]. Similarly, the acidity varies substantially among citric, malic, and tartaric acids. Therefore, the type, content, and relative proportion of organic acids and soluble sugars in mature fruits determine the taste of the fruit. Accordingly, optimization of the organic acids and soluble sugars compositions has become a primary objective of fruit tree breeding programs worldwide [5].

Apple (*Malus domestica* Borkh.) is an economically important fruit tree species in temperate regions, and its fruits contain many components that are beneficial for human health [6]. In apple fruit cells, most of the imported carbohydrates (sucrose and sorbitol) arrive and are released from the companion cell–sieve element complex of the phloem [7]. Sucrose transporter (SUT) and sorbitol transporter (SOT) are used to transport sucrose and sorbitol into the cytosol of parenchyma cells, respectively [7,8]. Additionally, cell wall invertase can cleave the sucrose into fructose and glucose, which are then transported into parenchyma cells based on the hexose transporter (HT) [9]. The soluble sugars entered into parenchyma cells are fed into the Suc-Suc cycle or undergo ‘futile Suc recycling’ [10]. The excess carbohydrates are converted into starch that is then stored in plastids or transported into vacuoles [7]. The fructose 6-phosphate (F6P) derived from sugar metabolism is used in glycolysis and in the tricarboxylic acid (TCA) cycle to produce energy and intermediates used in other processes [7]. Malic acid, which is the predominant organic acid in mature apple fruits, is mainly synthesized by malate dehydrogenase (MDH) in the cytosol and degraded by MDH and the NADP-malic enzyme (NADP-ME) [2]. Malic acid is also generated by the TCA cycle in the mitochondria and the glyoxylate cycle in glyoxysomes, in which NAD-MDH and malate synthase are the crucial enzymes controlling malate synthesis [11]. Malate can be degraded by two enzymes, namely NAD-ME in mitochondria and NADP-ME in chloroplasts or the cytoplasm [12]. Most organic acids and soluble sugars are stored in vacuoles [13]. The uptake of soluble sugars and organic acids by vacuoles requires many transporters and proton pumps located in the tonoplast, including the tonoplast sugar transporter (TST), vacuolar glucose transporter (vGT), and sugar will eventually be exported transporter (SWEET), SUT, aluminum-activated malate transporter (ALMT), P_3_-ATPase, and vacuolar ATPase (V-ATPase) [14]. The transient silencing of PpTST1 significantly decreased the sugar content of peach fruits [15]. The SUT, vGT, SWEET, and early response to dehydration 6-like transporter (ERDL6) genes also play roles in soluble sugar transport across the tonoplast [8,16,17,18]. Furthermore, genes controlling fruit acidity have been identified in apple including the *Ma1* [19,20], *SAUR*, *PP2C* [21], and *Ma10* [22]. There is increasing evidence that transcription factors (i.e., *MdMYB1* and *MdMYB73*) also contribute to the regulation of fruit acidity [23,24]. While study in fruit of these homologs first discovered in other species has begun, the mechanisms controlling organic acid and soluble sugar synthesis, degradation and accumulation in apple fruits is complex and remains unclear.

DNA methylation, which represents an epigenetic mark that is ubiquitous among eukaryotic species, can activate or silence gene transcription to regulate various processes [25]. In plants, DNA methylation occurs almost exclusively at cytosine residues [26]. Unlike in animals, where DNA methylation often occurs in the CG context, the methylation in plants occurs in the CG, CHG, and CHH (H = A, T, or C) contexts within various genomic regions [27]. There are three major DNA transferases that transfer a methyl group to cytosine: domain rearranged methyltransferase (DRM), methyltransferase 1 (MET1), and chromomethylase (CMT) [28]. The methylation of CG and CHG is carried out by MET1 and CMT3, respectively [29,30], whereas DRM2 and CMT2 are responsible for methylation of CHH [31]. De novo DNA methylation is mainly mediated by the RNA-directed DNA methylation (RdDM) pathways, including the canonical and noncanonical RdDM pathways in plants. The RdDM pathways are highly complex, requiring additional key factors, including RNA polymerase V and II [32]. During the plant life cycle, multiple processes are regulated by DNA methylation, including vegetative phase transitions [33], embryogenesis [34], fruit peel coloration, and anthocyanin biosynthesis [35,36]. However, little is known about methylation associated with organic acid and soluble sugar accumulation in apple fruits.

In this study, the transcriptome and DNA methylome were analyzed in conjunction with changes in the contents of soluble sugars and organic acids in the ‘Honeycrisp’ (HC) and ‘Qinguan’ (QG) apple cultivars. Analyses of global methylation and transcription revealed a correlation between gene expression levels and methylation states across the genomes. Two candidate genes, *MdMa11* and *MdTSTa*, were identified that influence organic acid and soluble sugar accumulation, respectively. The results revealed some of the complex gene network involved in the regulation of organic acid and soluble sugar accumulation in apple fruits.

## 2. Materials and Methods

### 2.1. Plant Materials and Treatment

Apple cultivars ‘HC’ and ’QG’ were grown at the Horticultural Experimental Station of Northwest A&F University (Xianyang, Shaanxi Province, China) with annual mean temperature 12.8 °C, average annual rainfall 699.4 mm and mean annual sunshine duration 1734.7 h. Mature fruits were collected at 90 days after full bloom (DAB) in 2019. Apple fruit maturity was determined based on the seed color and starch iodine test results [37]. Fruit samples were mechanically peeled, the fresh pulp was sliced into smaller pieces that were immediately frozen in liquid nitrogen and then stored at −75 °C.

### 2.2. Measurement of Organic Acid and Soluble Sugar Contents

The organic acid and soluble sugar contents were determined using an HPLC system according to previously reported [2,22]. All of the standards used in this study were purchased from Sigma Chemical Company (St. Louis, MO, USA) and then dissolved in ddH_2_O.

### 2.3. Bisulfite Sequencing (BS) Library Construction and Data Analysis

Genomic DNA was extracted from samples using a modified CTAB method. A total of 5.2 mg genomic DNA was mixed with 26 ng lambda DNA and then sonicated with the Covaris S220 ultrasonicator, which fragmented the DNA in to 200–300 bp segments. Cytosine-methylated barcodes were ligated to the sonicated DNA, after which the DNA fragments were treated twice with bisulfite using the EZ DNA Methylation-Gold kit (Zymo Research, Orange, CA, USA). The methylated cytosines would be unmodified, whereas the unmethylated cytosines were converted to uracil. A PCR was performed to construct the BS sequencing library. The insert size was estimated using the Agilent Bioanalyzer 2100 system (Agilent Technologies, Palo Alto, CA). Finally, the Illumina HiSeq 2500 platform (Novogene, Beijing, China) was used for the paired-end sequencing of the BS library.

The quality of the raw reads was evaluated using FastQC (version 0.11.5). After removing the low-quality reads and reads containing adapters from the raw reads, the remaining clean reads were aligned to the GDDH13 apple draft genome [38]. The bisulfite-converted versions (C-to-T and G-to-A) of the reference genome and sequencing reads were obtained in a directional manner, and the bisulfite-converted apple reference genome was indexed using Bowtie 2. The reads with a unique best alignment revealed by the two alignment processes were then compared with the non-converted genomic sequence. Based on these sequence comparisons, the methylated cytosine positions were determined.

To calculate the methylation level (ML), the reads were divided into multiple bins (10 kb), and the sum of read with and without methylation counts in each bin was calculated. The ML in each C site or bin was calculated using the following formula: ML (C) = reads (mC)/reads (mC + C). Furthermore, the calculated ML was corrected as follows: ML(corrected) = (ML − r)/(1 − r); r was considered as the bisulfite nonconversion rate as previously described [39].

The DSS software was applied to identify the differentially methylated regions (DMRs). A region at least three methylated cytosine sites in the CG, CHG, and CHH contexts were considered ‘methylated’ with a threshold less than 10−5. The bases exceeding 99.9% coverage were removed to minimize errors. The RepeatMasker program (http://www.repeatmasker.org/, 9 April 2019) was used to identify and annotate transposable elements (TEs) in the apple reference genome. Fisher’s exact test was used to detect the differentially methylated TEs.

### 2.4. RNA Sequencing (RNA-Seq) and Identification of Differentially Expressed Genes (DEGs)

The NEBNext^®^ Ultra™ RNA Library Prep Kit for Illumina^®^ (NEB, Ipswich, MA, USA) was used to construct RNA-seq libraries from 1 μg RNA. The libraries were sequenced using the Illumina HiSeq 2000 platform (Illumina, San Diego, CA, USA) to produce 150-bp paired-end reads. Clean reads were obtained after removing low-quality reads as well as reads with adapters and poly-N sequences from the raw reads. The HISAT2 (version 2.0.5) software was used for indexing the apple draft genome (GDDH13, https://iris.angers.inra.fr/gddh13/) and for aligning the paired-end clean reads to the apple draft genome.

Gene expression levels were estimated according to the fragments per kilobase of transcript per million mapped reads (FPKM) values. The DEGSeq2 R package was performed to analyze differential gene expression. The adjusted *p*-value was used to control the false discovery rate (FDR).

### 2.5. Subcellular Localization of MdTSTa and MdMa11 in N. benthamiana Leaf Epidermal Cells

The full *MdTSTa* and *MdMa11* coding sequences were amplified from the cDNA template derived from ‘HC’ fruits, and then inserted into the pMDC83 vector for the subsequent expression under the control of dual cauliflower mosaic virus (CaMV) 35S promoters. The resulting constructs for the constitutive expression of the MdTSTa-GFP and MdMa11-GFP fusion proteins were separately introduced into cells of *Agrobacterium tumefaciens* strain GV3101 via electroporation, and then transiently transformed into *N. benthamiana* leaves by infiltration of *A. tumefaciens* [20]. The infiltrated plants were cultivated in a growth chamber at 24 °C with a 16-h light/8-h dark cycle. Vacuoles were extracted from the transformed leaves. The GFP signal and chlorophyll autofluorescence were detected at 488 and 750 nm, respectively, using a Leica TCS SP8 confocal laser scanning microscope (Leica, Weltzlar, Germany).

### 2.6. Functional Analysis of MdTSTa and MdMa11

The full *MdTSTa* coding sequence was inserted into the pHellsgate 2 expression vector. The pHellsgate 2-MdTSTa recombinant plasmid was inserted into cells of *A. tumefaciens* strain EHA105 via electroporation, and then transformed into the tomato (*Solanum lycopersicum* L.) cultivar ‘Micro-Tom’ using by leaf disc infiltration [18]. The transgenic plants were verified based on their kanamycin resistance and PCR sequencing analysis. Mature fruits were randomly harvested from each line, with three replicates comprising approximately 10 fruits each. Fruit juice was collected, filtered through gauze, and centrifuged at 5000 × *g* for 15 min. The supernatants were used to determine the fruit soluble sugar contents. The full *MdMa11* coding sequence was inserted into cells of the pMDC83 expression vector. The pMDC83-MdMa11 recombinant plasmid was inserted into *A. tumefaciens* strain GV3101 via electroporation for the transient transformation of tobacco described by Wang et al. [40]. Details regarding the primers used in this study are listed in Table S1.

## 3. Results

### 3.1. Phenotypic Difference between ‘HC’ and ‘QG’ Mature Apple Fruits

**Figure 1:**
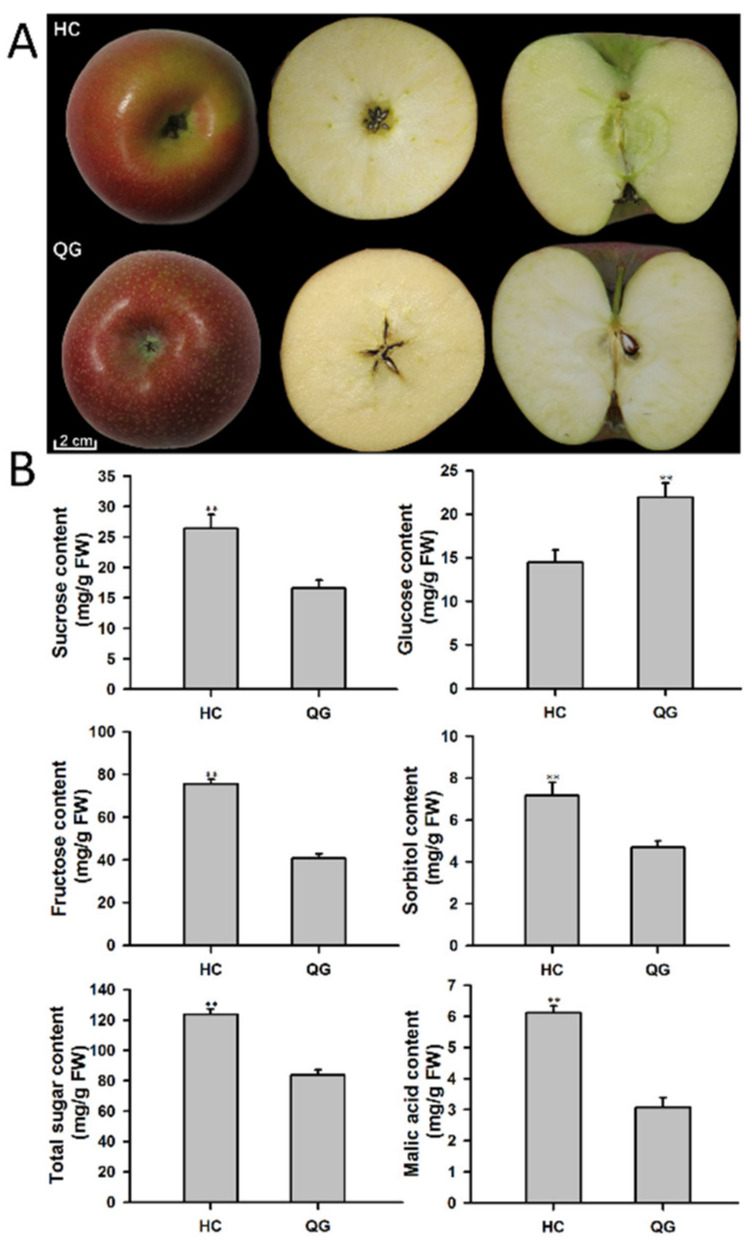
Phenotypes of ‘Honeycrisp’ (‘HC’) and ‘Qinguan’ (‘QG’) mature fruits. (**A**) Morphological characteristics of ‘HC’ and ‘QG’ mature fruits. (**B**) Soluble sugar and malic acid contents of ‘HC’ and ‘QG’ mature fruits. Double asterisks (**) represent significant differences between ‘HC’ and ‘QG’ mature fruits (*t*-test, *p* < 0.01).

Mature ‘HC’ and ‘QG’ apple fruits were harvested at 90 DAB. The phenotypic characteristics of the fruits are presented in Figure 1. The ‘HC’ and ‘QG’ mature fruits were similar in size, including the longitudinal and transverse diameters (Figure 1A). However, there were significant differences in the organic acids and soluble sugars contents (Figure 1B). The fructose, sucrose, sorbitol, and total sugar contents were higher in ‘HC’ mature fruits than in ‘QG’ mature fruits. Additionally, ‘HC’ mature fruits were significantly more acidic than ‘QG’ mature fruits, with the average malic acid content of ‘HC’ mature fruits more than 1.9-times greater than that of ‘QG’ mature fruits.

### 3.2. Genome-Wide Methylation Profiles in ‘HC’ and ‘QG’ Mature Fruits

To investigate the methylation patterns in ‘HC’ and ‘QG’ mature fruits, Bisulfite sequencing (BS) libraries were constructed and sequenced using the Illumina HiSeq 2500 platform. A total of 109,036,039 and 83,861,672 raw reads (approximately 32.71 and 25.16 Gb of data) were obtained for the ‘HC’ and ‘QG’ mature fruits, respectively (Table S2). After removing reads with low-quality and the adapters, 107,405,734 and 82,887,115 clean reads (approximately 29.52 and 22.77 Gb of data) remained for the ‘HC’ and ‘QG’ mature fruits, respectively. The datasets provided more than 20× deep coverage and a 99.8% bisulfite conversion rate. 

Approximately 90.64% and 93.65% of the sequenced bases had a quality score greater than Q30. The GC contents of the sequenced bases for ‘HC’ and ‘QG’ were 23.03% and 23.87%, respectively (Table S2). Of the clean reads from ‘HC’ and ‘QG’ mature fruits, 46.47% (49,911,444) and 56.22% (46,599,136) respectively, were uniquely aligned to the GDDH13 apple draft genome. Details regarding genome coverage, the accumulative fraction of CX (%), the depth and proportion of covered bases, and the insert size distribution are presented in Table S3 and Figure S1.

The whole-genome DNA methylation patterns in CG, CHH and CHG context were analyzed for the ‘HC’ and ’QG’ mature fruits (Figure S2). The chromosomal distribution of each context, the density of TEs or mClevels, and gene or mC densities for each chromosome were evaluated (Figures S2A,B and S3A,B). Circos plots revealed significant differences in various chromosomal regions (Figures S2A,B and S3A,B). The estimated methylation rates in each context revealed that the methylation rate was higher for CHH (61.04% and 55.59% in ‘HC’ and ‘QG’ mature fruits, respectively) than for CG (21.07% and 24.13% in ‘HC’ and ‘QG’ mature fruits, respectively) or CHG (17.88% and 20.27% in ‘HC’ and ‘QG’ mature fruits, respectively) (Figure S2C). Additional details regarding the methylation rates and distribution of methylation levels in each context among the chromosomes in ‘HC’ and ‘QG’ mature fruits are presented in Figures S2D and S4A,B. An analysis of the methylation level and density in all three contexts indicated that, in both ‘HC’ and ‘QG’ mature fruits, the CG context had the highest methylation level (48.12% and 50.69% for ‘HC’ and ‘QG’, respectively), and then the CHG context (32.74% and 35.55% for ‘HC’ and ‘QG’, respectively). The mean methylation level in the CHH context was lowest (9.84% and 11.10% for ‘HC’ and ‘QG’, respectively) (Figures S2E and S4C). Similar variations were detected for the methylation density (Figures S2E and S4D).

### 3.3. Distribution of DNA Methylation among Gene Features and TEs

**Figure 2:**
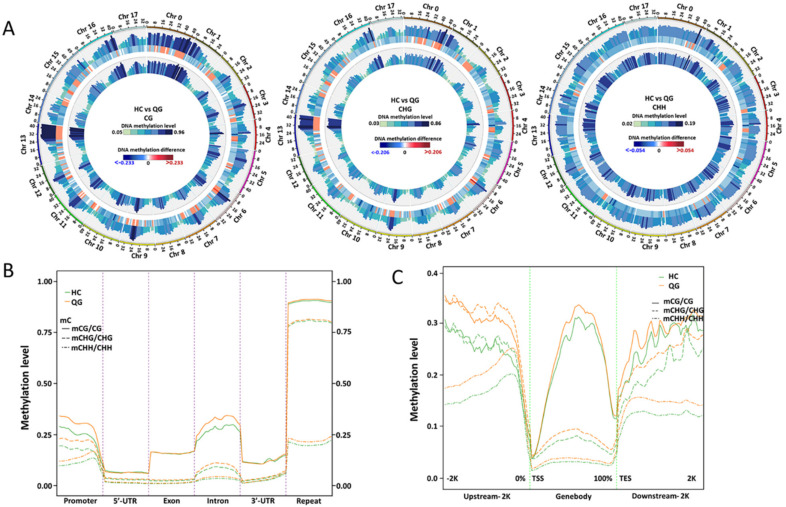
Comparison of the methylation in the CG, CHG, and CHH contexts in various genomic regions between ‘HC’ and ‘QG’ mature fruits. (**A**) Circos plots comparing the DNA methylation levels between ‘HC’ and ‘QG’ mature fruits. (**B**) Comparison of the DNA methylation patterns in various genomic regions between the mature fruits of ‘HC’ (green lines) and ‘QG’ (orange lines). (**C**) Comparison of the methylation levels in the upstream or downstream regions and gene bodies between the mature fruits of ‘HC’ (green lines) and ‘QG’ (orange lines). Solid lines, mCG; dash lines, mCHG; mixed line, mCHH. TSS, transcriptional start site; TES, transcriptional end site.

The distribution of DNA methylation was analyzed in the ‘HC’ and ‘QG’ mature fruits. Significant differences were detected among gene features [i.e., promoters, introns, exons, 3′ and 5′untranslated regions (UTRs), and repeats] based on a heat map analysis (Figure S2F). The DNA methylation levels in CG, CHG, and CHH context were highest in the repeat regions, and then in the promoter, intron, and exon regions (Figures S2F and S5A,B). The DNA methylation levels in both CHG and CHH contexts were significantly lower in the gene bodies than that in 2 kb downstream and upstream regions of gene bodies in both fruit (Figure S5C,D). The differences in the DNA methylation between ‘HC’ and ‘QG’ mature fruits are presented in Figure 2. Circos plots revealed significant differences between ‘HC’ and ‘QG’ mature fruits in all three contexts in various regions (Figure 2A). Interestingly, the DNA methylation levels in all three contexts in the promoters were significantly higher in ‘HC’ than in ‘QG’ (Figure 2B). Similar results were observed for the CHH context in the repeat regions as well as for the CHG and CG contexts in the intron (Figure 2B). Furthermore, the DNA methylation levels in each context in upstream region of the transcriptional start site (TSS) and downstream region of the transcriptional termination site (TTS) and in gene bodies were higher in ‘HC’ mature fruits than in ‘QG’ mature fruits (Figure 2C), especially in the CHH context in downstream region of the TTS and upstream region of the TSS and in the CHG context in gene bodies (Figure 2C).

The methylation levels of TEs, including DNA, helitron, long interspersed nuclear element (LINE), and long terminal repeat (LTR) sequences, were significantly different between various regions (i.e., gene body, downstream and upstream regions) (Figure S6). The methylation levels in the CG and CHG contexts in the DNA, LTR and LINE TEs in the 2-kb flanking upstream and downstream regions were significantly higher in the ‘QG’ mature fruits than in the ‘HC’ mature fruits. The methylation levels in the CHG and CG contexts in helitron TEs in the 2-kb flanking regions and the gene bodies were significantly higher in the ‘QG’ mature fruits than in the ‘HC’ mature fruits (Figure S6). The methylation levels in the CHH context in all TEs were significantly higher in ‘QG’ mature fruits than in ‘HC’ mature fruits (Figure S6).

### 3.4. Identification and Analyses of the Distribution and Functions of the Differentially Methylated Regions (DMRs) between ‘HC’ and ‘QG’ Mature Fruits

**Figure 3:**
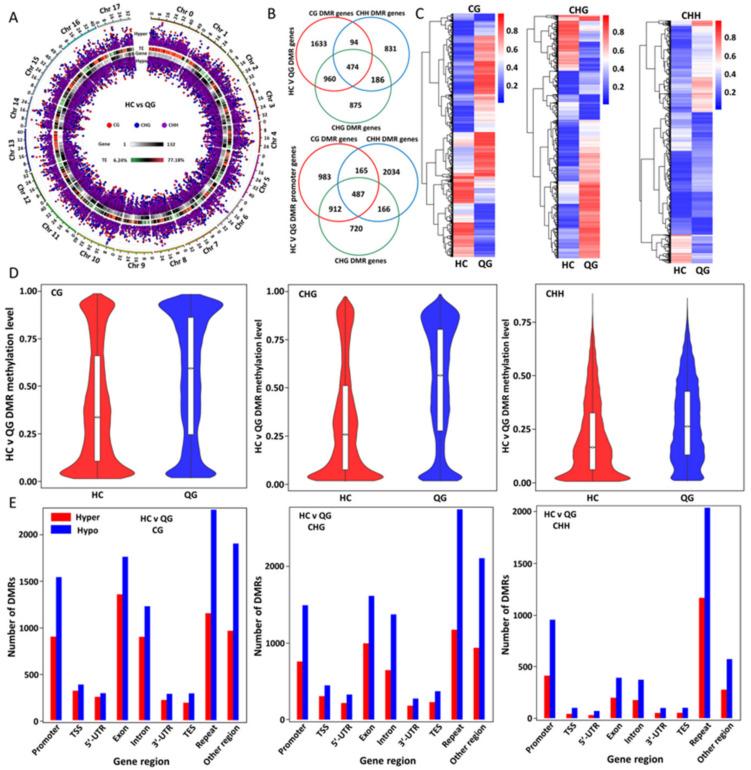
DMRs between ‘HC’ and ‘QG’ mature fruts. (**A**) Circos plot presenting the hyper- and hypo-DMRs in the CG, CHH, and CHG contexts between ‘HC’ and ‘QG’ mature fruits. (**B**) Venn diagrams of the DMRsin genes and DMRsin promoters between ‘HC’ and ‘QG’ mature fruits. DMRs in the center would contain all three methylation types (CG, CHH, and CHG). (**C**) Heat maps clustering the DMRs in the CG, CHH, and CHG contexts between ‘HC’ and ‘QG’ mature fruits. (**D**) Violin plots comparing the DMRs in the CG, CHH, and CHG contexts between ‘HC’ and ‘QG’ mature fruits. (**E**) Number of DMRs in the CG, CHH, and CHG contexts in various genomic regions in ‘HC’ and ‘QG’ mature fruits.

The DMRs were identified based on a comparison of the DNA methylation levels between ‘HC’ and ‘QG’ mature fruits (‘HC’ vs ‘QG’) (Figure 3 and Figure S7). The distribution of DNA methylation in all three contexts in the ‘HC’ vs ‘QG’ comparison (Figure 3A and Figure S3C) exhibited significant differences among the whole genome. Venn diagrams were constructed to present the distribution of DMRs containing ‘HC’ vs ‘QG’ DMR genes and promoters in all three contexts (Figure 3B). For the CG, CHG, and CHH contexts, a total of 3161, 1585, and 2495 DMRs in genes and 2547, 2852, and 2285 DMR promoters, respectively, were identified in the ‘HC’ vs ‘QG’ comparisons. The DMRs and their methylation levels in the CG, CHH and CHG contexts (FDR < 0.05) for the ‘HC’ vs ‘QG’ comparison group were visualized as heat maps (Figure 3C). The methylation levels in all three contexts in the DMRs were significantly higher in ‘HC’ mature fruits than in ‘QG’ mature fruits (Figure 3D). An examination of the DMRs in all three contexts in various genic regions indicated that the number of DMRs in the CG, CHH and CHG contexts differed significantly in the promoter, TSS, TTS, exon, repeat, intron, 3′/5′-UTR, and other regions (Figure 3E). In all genic regions, fewer hypermethylated DMRs than hypomethylated DMRs in all three contexts were observed. There were more DMRs (hypomethylated/hypermethylated) in the CG, CHH, and CHG contexts in the TSS, TTS, and 3′/5′-UTR regions than in the other examined regions.

### 3.5. Analysis of the Association between the Transcriptome and DNA Methylation

To elucidate the relationship between DNA methylation and gene expression in mature apple fruits, the correlation between gene expression levels and the methylation levels in all three contexts was analyzed. The fruit transcriptome analysis was completed using the same ‘HC’ and ‘QG’ mature fruits that were used for the BS sequencing. Details regarding the RNA-seq data for the ‘HC’ and ‘QG’ mature apple fruits are listed in Table S4. Heat maps were constructed for the differentially expressed genes (DEGs) between ‘HC’ and ‘QG’ mature apple fruits (Figure S8A). A total of 22,657 (11,219 upregulated and 11,438 downregulated) DEGs in the ‘HC’ vs ‘QG’ comparison group were identified (*p* < 0.05) (Figure S8B). Circos plots of the genome-wide methylation intensities and distribution of the whole transcriptome reads revealed significant differences between ‘HC’ and ‘QG’ mature apple fruits (Figure S8C,D). The correlation between gene expression and DMR methylation levels was analyzed (Figures S9 and S10). A negative correlation was detected in gene body and promoter DMRs in all three contexts (Figure S9A–J). In contrast, a positive correlation was detected in promoter DMRs in the CHH context (Figure S9K,L). Furthermore, the gene expression levels were divided into the quartiles according to their FPKM values (Figure S10A). High gene expression levels were accompanied by low methylation levels in the CG and CHG contexts in the regions downstream of the TTS as well as in the CHG and CHH contexts in the gene bodies in both the ‘HC’ and ‘QG’ mature fruits (Figure S10A). However, the genes with high methylation levels were the most highly expressed genes in the CG context in the downstream regions of the TTS and in the CHH context in the promoter regions (upstream of the TSS) in both the ‘HC’ and ‘QG’ mature fruits. Interestingly, the genes with the least methylation levels accompanied by the lowest expression levels in the CG context in the gene body region in both ‘HC’ and ‘QG’ mature fruits (Figure S10A).

To further investigate the correlation between gene expression and methylation levels, the methylated genes were divided into the five groups according to the methylation levels of promoter and gene body (Figure S10B). The genes with the highest methylation levels (group 5) in the CHG and CHH contexts (the bottom two rows of panels) in the gene body regions (left two panels) had the lowest expression levels in both the ‘HC’ and ‘QG’ mature fruits, and the genes with the lowest methylation levels in the CG context in gene body regions (group 1) had the lowest expression levels in both the ‘HC’ and ‘QG’ mature fruits (Figure S10B). This indicated that the methylation level within genic regions in the CHH and CHG contexts was negatively correlated with gene expression level, whereas methylation level of gene body regions in the CG context was positively correlated with gene expression level. A similar finding was obtained for the CHG and CG contexts in the promoter regions. Thus, the gene expression and methylation levels were inversely related in both the ‘HC’ and ‘QG’ mature fruits (Figure S10B). In contrast, genes which contained highest methylation levels in the promoter regions in the CHH context (group 5), had the highest expression levels, implying the gene expression level is positively correlated with methylation level in promoter regions.

### 3.6. Differences in the Expression and Methylation Levels of Genes Involved in Soluble Sugar and Organic Acid Metabolism between ‘HC’ and ‘QG’ Mature Fruits

To clarify the mechanisms underlying the organic acids and soluble sugars metabolism in ‘HC’ and ‘QG’ mature fruits, the methylation and expression levels of related genes were analyzed. The expression of 91 analyzed genes differed significantly (Figure S11A). The expression levels of sugar metabolism-related genes (e.g., cell wall invertase and fructokinase genes) were significantly higher in ‘QG’ mature fruits than in ‘HC’ mature fruits (Figure S11A, Table S5). Many sugar transporters (e.g., SWEET and HT genes) were also higher expressed in ‘QG’ mature fruits than in ‘MC’ mature fruits. The expression levels of other sugar metabolism-related genes and sugar transporter genes, including those encoding a sucrose synthase, hexokinase, sorbitol dehydrogenase, SUT, and SOT, also differed between ‘HC’ and ‘QG’ mature fruits (Figure S11A, Table S5). There were 32 genes related to organic acid metabolism (four cytMDH, four mitMDH, four mitME, three PDH, two CS, three ACO, and three IDH genes) expressed at lower levels in ‘HC’ mature fruits than in ‘QG’ mature fruits. However, the opposite pattern of expression was detected for four cytME genes. Furthermore, six phosphoenolpyruvate carboxykinase (PEPC) genes were differentially expressed between ‘HC’ and ‘QG’ mature fruits. Among these six PEPC genes, lower expression levels of one PEPC3 and three PEPC1 genes in ‘HC’ mature fruits than in ‘QG’ mature fruits were observed, whereas two PEPC4 genes were lower expressed in mature fruits of ‘QG’ than of ‘HC’. Similar results were obtained for ATP-citratelyase (ACL) and chlME genes. Additionally, one gene encoding a plasma membrane-localized ALMT and four genes encoding a chloroplast membrane-localized ATPase were expressed at higher levels in ‘QG’ mature fruits than in ‘HC’ mature fruits (Figure S11A, Table S5). Higher methylation levels in the CHH context in promoters and in the CG context in gene bodies were positively associated with gene expression in ‘HC’ and ‘QG’ mature fruits (Figure S11B–D). However, the methylation levels in the CHH context in introns were negatively associated with gene expression in ‘HC’ and ‘QG’ mature fruits (Figure S11C).

### 3.7. Vacuolar Transporter Genes: Differences in the Expression and Methylation Levels between ‘HC’ and ‘QG’ Mature Fruits

**Figure 4:**
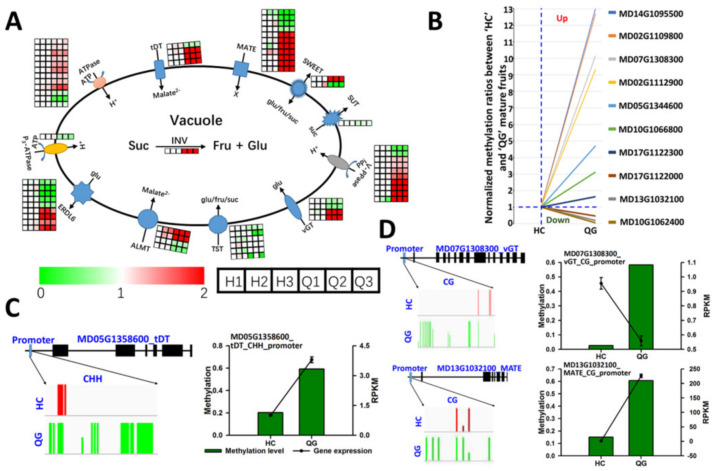
Expression and methylation levels of genes involved in soluble sugar and organic acid transport into vacuoles. (**A**) Differentially expressed genes involved in organic acid and soluble sugar transport between ‘HC’ (left 3 boxes) and ‘QG’ (right 3boxes) mature fruits. (**B**) Cluster analysis of differentially methylated genes. (**C**,**D**) IGV snapshots of DNA methylation and gene expression in ‘HC’ and ‘QG’ mature fruits.

Organic acids and soluble sugars in fruit cells accumulate in vacuoles. The vacuolar storage of organic acids and soluble sugars requires many transporters and proton pumps in the tonoplast [e.g., SWEET, ALMT, V-ATPase, AVA, TST, vGT, SUT, and tonoplast dicarboxylate transporter. A total of 64 genes related to soluble sugar transporters, organic acid transporters, and proton pumps were differentially expressed between ‘HC’ and ‘QG’ mature fruits (Figure 4A). Among these 64 DEGs, all six TST-encoding genes involved in sucrose, fructose, and glucose transport into vacuoles were more highly expressed in ‘HC’ mature fruits than in ‘QG’ mature fruits. The SUT and acid invertase genes were also expressed at higher levels in ‘HC’ mature fruits than in ‘QG’ mature fruits. Additionally, two vGT genes, one SWEET gene, and four ERDL6 genes were more highly expressed in ‘HC’ mature fruits than in ‘QG’ mature fruits, whereas the opposite patterns were detected for two vGT genes, one SWEET gene, and five ERDL6 genes. Furthermore, 31 DEGs related to organic acid transport and proton pumps were identified, of which one gene (MD17G1155800) encoding a P_3A_-ATPase (designated as *Ma11*) and *Ma1* gene were expressed at significantly higher levels in ‘HC’ mature fruits than in ‘QG’ mature fruits. Previous research confirmed *Ma1* as a major gene controlling fruit acidity (Figure 4A) [19,20]. The methylation levels of seven transporter and proton pump genes were significantly lower in ‘HC’ mature fruits than in ‘QG’ mature fruits (Figure 4B), which contributed to the higher expression levels of these genes in ‘HC’ mature fruits than in ‘QG’ mature fruits (Table S5). The opposite patterns of methylation and expression were detected for three other genes (Figure 4B, Table S5). The examination of the association between methylation levels in each context (CG, CHH, and CHG) and gene expression levels indicated that high methylation levels in promoters in the CHH context was positively associated with gene expression levels (Figure 4C). However, the methylation level in the CG context in promoters was not associated with gene expression (Figure 4D).

### 3.8. Identification and Functional Analysis of Genes Involved in Soluble Sugar Accumulation

**Figure 5:**
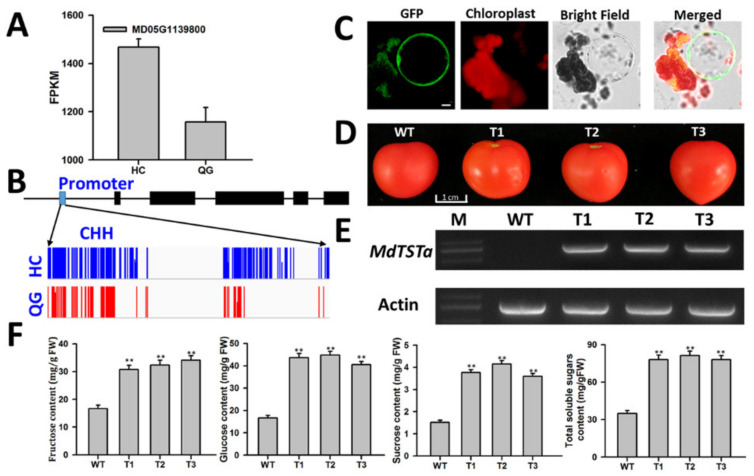
Functional characterization of *MdTSTa*. (**A**) *MdTSTa* expression levels in ‘HC’ and ‘QG’ mature fruits. (**B**) Differences in the methylation level of the *MdTSTa* promoter in the CHH context between ‘HC’ and ‘QG’ mature fruits. (**C**) Tonoplast localization of the MdTSTa-GFP fusion protein in vacuoles, which were obtained after transiently transformed *N. benthamiana* protoplasts were lysed. Bars represent 10 μm. (**D**) Mature tomato fruits from wild-type and 35S: *MdTSTa* transgenic lines. (**E**) *MdTSTa* expression in wild-type and transgenic tomato based on RT-PCR data. (**F**) Soluble sugar contents in the mature tomato fruits of wild-type and transgenic lines. WT: wild-type; T1, T2, and T3: 35S: *MdTSTa* transgenic lines. Double asterisks (**) represent significant differences between wild-type and transgenic fruits (*t*-test, *p* < 0.01).

Because soluble sugars are mainly stored in vacuoles, we focused on identifying candidate genes encoding tonoplast-localized proteins that are expressed in both ‘HC’ and ‘QG’ mature fruits, but at significantly different levels. Among the DEGs involved in the transport of soluble sugars across the tonoplast, one TST gene (*MdTSTa*; MD05G1139800) was expressed in both ‘HC’ and ‘QG’ mature fruits, but at significantly different levels (Figure 5A). The methylation levels of the *MdTSTa* promoter and gene body were analyzed. The methylation level in the CHH context in the *MdTSTa* promoter was higher in ‘HC’ mature fruits than in ‘QG’ mature fruits (Figure 5B). To determine the subcellular localization of MdTSTa, the MdTSTa-GFP fusion construct was expressed in *N. benthamiana* leaves. Protoplasts were isolated from the transiently transformed leaves and then lysed to release the intact vacuoles (Figure 5C). The MdTSTa-GFP fusion proteins were detected in the tonoplast (Figure 5C). The effects of MdTSTa overexpression (OE) on the soluble sugar concentration were evaluated in transgenic tomato plants. The fruits of three MdTSTa-OE lines were similar in size to the wild-type fruits (Figure 5D). An RT-PCR analysis indicated that *MdTSTa* was highly expressed in the transgenic fruits (Figure 5E). The soluble sugar contents, including glucose, fructose, and sucrose, in the wild-type and transgenic fruits are presented in Figure 5F. The average sucrose, fructose, glucose, and total soluble sugar contents were significantly higher in the transgenic fruits than in the wild-type fruits (Figure 5F). These observations suggested that *MdTSTa* is important for the accumulation of soluble sugars in fruits.

### 3.9. Identification of a Candidate Gene Affecting Vacuolar Acidity

**Figure 6:**
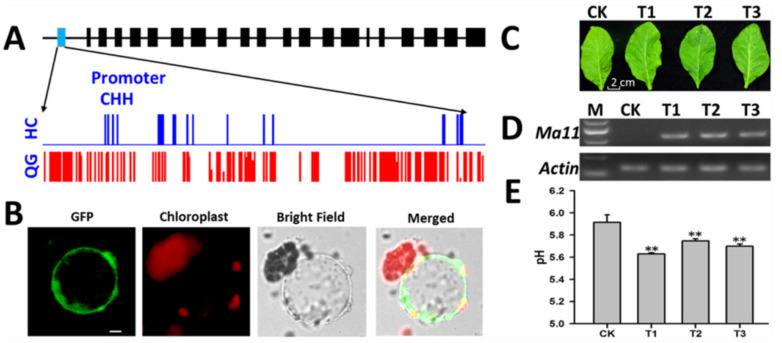
Functional characterization of *MdMa11*. (**A**) Differences in the methylation of the *MdMa11* promoter in the CHH context between ‘HC’ and ‘QG’ mature fruits. (**B**) Tonoplast localization of the MdMa11-GFP fusion protein in vacuoles, which were obtained after transiently transformed *N. benthamiana* protoplasts were lysed. Bars represent 10 μm. (**C**) Transiently transformed tobacco leaves. CK represents tobacco leaves transformed with the empty vector, whereas T1, T2, and T3 represent tobacco leaves transformed with *MdMa11*. (**D**) Analysis of *MdMa11* expression in tobacco leaves transiently transformed with the empty vector (CK) or *MdMa11* (T1, T2, and T3) based on RT-PCR data. (**E**) Analysis of the pH of tobacco leaves transiently transformed with the empty vector (CK) or *MdMa11* (T1, T2, and T3). Double asterisks (**) indicate significant differences in the pH of tobacco leaves transiently transformed with the empty vector (CK) or *MdMa11* (T1, T2, and T3) (*t*-test, *p* < 0.01).

As mentioned above, one gene (*MdMa11*) encoding a P_3A_-ATPase exhibited significantly higher gene expression levels in ‘HC’ mature fruits than in ‘QG’ mature fruits (Table S5). The DNA methylation levels in the CG, CHH, and CHG contexts in different *MdMa11* genomic regions were investigated. The methylation levels in the CHH context in the MdMa11 promoter were higher in ‘QG’ mature fruits than in ‘HC’ mature fruits (Figure 6A). During the examination of the subcellular localization of MdMa11, the MdMa11-GFP fusion protein was detected in the tonoplast (Figure 6B). To determine whether *MdMa11* overexpression affects vacuolar acidity, *MdMa11* was transiently expressed in tobacco leaves. No significant morphological differences were observed between wild-type and transiently transformed tobacco leaves (Figure 6C). The results of an RT-PCR analysis confirmed that *MdMa11* was highly expressed in the transiently transformed tobacco leaves (Figure 6D). The pH values of the tobacco leaves transiently transformed with *MdMa11* or the empty vector were measured (Figure 6E). The average pH of the tobacco leaves transiently transformed with *MdMa11* was 5.69, which was significantly lower than that in the tobacco leaves carrying the empty vector (5.92). These results suggested that *MdMa11* has functions that modulate vacuolar acidity.

## 4. Discussion

Apple is an important fruit crop which widely cultivated in temperate regions worldwide. Moreover, apple fruits are very popular among consumers because of their high organoleptic quality, which is mainly influenced by soluble sugars and organic acids [1,2,41]. For many biological processes, DNA methylation is critical, and disruptions to DNA methylation can lead to abnormal phenotypes in plants [38]. The methylation of DNA at the 5′ position of cytosines is the predominant epigenetic modification regulating nuclear gene expression and genome stability [42]. Therefore, the epigenetic modifications in apple fruits with different organic acid and soluble sugar contents were investigated to clarify the epigenetic regulation of organic acid and soluble sugar metabolism and to identify the specific mechanism underlying these fruit traits.

Because of the advances in high-throughput sequencing technology, whole-genome BS has become a particularly powerful method for elucidating novel regulatory mechanisms mediating various biological processes, including fruit ripening, imprinting, seed development, and responses to environmental stimuli [42,43]. However, the epigenetic regulation of organic acid and soluble sugar metabolism in apple fruits remains unclear. In the current study, we conducted a genome-wide analysis of the DNA methylation in ‘HC’ and ‘QG’ mature fruits and sought epigenetic variations between the two cultivars. The methylation levels were approximately 49%, 34%, and 10% in the CG, CHG, and CHH contexts, respectively (Figure S2E). These results were consistent with those of earlier investigations on rice, soybean, poplar, and *Arabidopsis thaliana* [33]. The highest relative proportion of mCs was observed in the CHH context (Figure S2C), which is in accordance with the findings of previous studies on poplar and birch, but differs from the results of earlier research involving *A. thaliana* and rice, in which the mCs ratio was highest in the CG context [44]. Genomic stability may decrease because of the new copies of retrotransposons insertion or the DNA transposons relocation. In the *A. thaliana* genome, TE-containing regions are heavily methylated in CG, CHG, and CHH contexts [42,45]. Similarly, in the current study, the methylation levels in all three contexts were positively related with the TE density, but negatively related with gene abundance (Figure S2A) [43]. These results imply that DNA methylation sites mainly contribute to the maintenance of genome stability. 

In plants, the precise molecular mechanisms underlying transcriptional control have not been comprehensively characterized, but the regulation of gene expression by DNA methylation in various regions has been investigated [36]. The methylation of promoters usually inhibits transcription. A recent genome-wide analysis of methylation revealed the complexity of the relationship between DNA methylation in promoters and gene expression [42]. In our study, the promoter methylation in the CHG and CG contexts was usually negatively related with gene expression, in contrast to the positive correlation between gene expression and the promoter methylation in the CHH context (Figure S9). Presumably, the promoter methylation directly represses transcription by inhibiting the binding of transcriptional activators or by promoting the binding of transcriptional repressors, and indirectly represses transcription by regulating the histone modifications [42,46]. It remains unknown how promoter methylations promote gene expression. Furthermore, the methylation of gene bodies was thoroughly investigated in the apple fruits. In contrast to transposons, promoters, and repeating sequences, which are highly methylated in all three contexts (CG, CHH, and CHG), we observed that gene bodies were mainly methylated in the CG context (Figure 2B, Figures S6 and S10A), which is consistent with the previous finding [47]. The negative correlation between the methylation of gene bodies and gene expression was revealed in this study (Figure S9) as well as in a recent study [33]. However, earlier research suggested that methylated gene bodies are expressed at higher levels than unmethylated gene bodies in *A. thaliana* [45]. This discrepancy might be because of species-specific gene body methylations.

Apple fruit acidity and sweetness are complex quantitative traits that are determined by organic acid and soluble sugar contents, respectively. In the current study, the methylation and expression of genes involved in organic acid and soluble sugar metabolism and transport were investigated (Figure 4, Figure S11, Table S5). Some genes were associated with hyper- and hypo- methylated regions, and many downregulated and upregulated genes varied considerably in terms of methylation levels (Figure 4D), implying complicated regulation of gene expression by DNA methylation. Earlier research proved that the overexpression of TST genes can increase the accumulation of sugars in vacuoles [15,48]. In our investigation, the overexpression of one candidate gene (*MdTSTa*) increased the fructose, glucose, sucrose, and total soluble sugar contents in tomato fruits (Figure 5). In mature fruits, the methylation level in the CHH context in the *MdTSTa* promoter was higher in ‘HC’ than in ‘QG’ fruits, indicating that *MdTSTa* modulates the apple fruit soluble sugar content via DNA methylation changes. The predominant organic acid in apple fruits is malic acid, which it is mainly stored in vacuoles [2,49]. The ‘acid trap’ mechanism enables the accumulation and maintenance of malic acid in vacuoles in apple fruit cells [11,22]. The expression of the *Ma1* gene reportedly increases the uptake of malate by vacuoles, thereby influencing the acidity of mature apple fruits [19,20]. Gene expression analysis revealed that *Ma1* was more highly expressed in ‘HC’ mature fruits than in ‘QG’ mature fruits, indicating that more malate was transported into the vacuoles of ‘HC’ mature fruit cells than into the vacuoles of ‘QG’ mature fruit cells. To maintain the electroneutral state of vacuoles, cations (e.g., H+ or mineral cations) should be transported into vacuoles. In the current study, a gene encoding a tonoplast-localized P_3A_-ATPase (*MdMa11*, which is homologous to *Ma10*) was confirmed to influence apple fruit acidity (Figure 6). The methylation level in the CHH context in the *MdMa11* promoter was lower in ‘HC’ mature fruits than in ‘QG’ mature fruits, which is consistent with the findings of a recent study [36]. However, inconsistent results were obtained for *MdTSTa* (Figure 5, Figure S9K–L). This discrepancy might be related to the precise molecular mechanisms underlying the transcriptional control resulting from DNA methylations. The regulation of gene expression by DNA methylation may occur via the methylation of the gene itself (cis-element) and/or the methylation of a distal genomic site [50].

**Figure 7:**
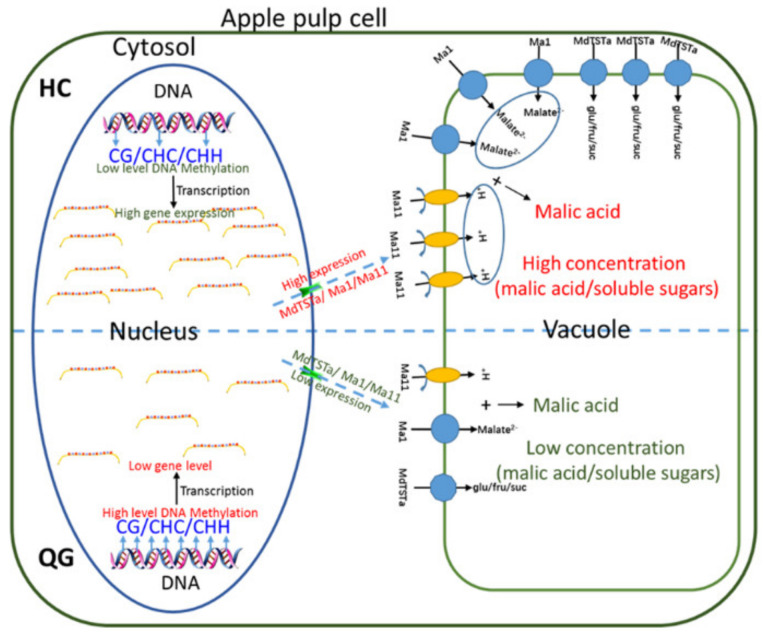
Proposed model for the epigenetic regulation of organic acid and soluble sugar in ‘HC’ and ‘QG’ mature fruits. Methylation levels are higher in ‘QG’ mature fruits than in ‘HC’ mature fruits. Additionally, MdTSTa mediates the transport of soluble sugars from the cytosol into the vacuole, whicle MdMa1 and MdMa11 transport malic acid and protons into the vacuole, respectively. Upregulated MdTSTa, MdMa1, and MdMa11 expression levels tend to result in higher vacuolar soluble sugar and malic acid concentrations in ‘HC’ mature fruits than in ‘QG’ mature fruits.

In summary, the data presented herein confirmed the importance of epigenetic modifications for apple fruit soluble sugar and organic acid accumulation. Moreover, we demonstrated that overexpressing *MdTSTa* and *MdMa11* can increase the soluble sugars concentration and vacuolar acidity, respectively (Figure 5 and Figure 6). Earlier studies proved that the *Ma1* gene encodes a tonoplast-localized protein that is significantly associated with the malic acid content (i.e., fruit acidity). Thus, we propose that soluble sugars and organic acids accumulate in vacuoles via *MdTSTa* and the interaction between *MdMa11* and *MdMa1* (Figure 7). The results form the basis of future studies aimed at comprehensively characterizing the complex mechanism regulating apple fruit acidity.

## Data Availability

The datasets generated and/or analyzed during the current study are available from the corresponding author on reasonable request.

## Supplementary Materials

The following are available online at https://www.mdpi.com/article/10.3390/foods10092198/s1, Figure S1. Characterization of bisulfite sequencing data in ‘HC’ and ‘QG’ mature fruits; Figure S2. Details regarding the ‘HC’ and ‘QG’ mature fruit epigenomes; Figure S3. Detail information of epigenome in ‘HC’ and ‘QG’ mature fruits; Figure S4. Methylation patterns of CG, CHG and CHH contexts in ‘HC’ and ‘QG’ mature fruits; Figure S5. DNA methylation pattern in different genomic regions in ‘HC’ and ‘QG’ mature fruits; Figure S6. Methylation patterns of TEs in ‘HC’ and ‘QG’ mature fruits. U and D: 2 kb upstream and downstream regions, respectively; G: gene body regions; Figure S7. Distribution of DMR lengths in the CG, CHG, and CHH contexts in ‘HC’ and ‘QG’ mature fruits; Figure S8. Differentially expressed genes (DEGs) between ‘HC’ and ‘QG’ mature fruits; Figure S9. Association analysis between DNA methylation levels and gene expression of CH, CHG and CHH contexts in DMR gene bodies and DMR promoters between ‘HC’ and ‘QG’ mature fruits; Figure S10. Correlation between DNA methylation and gene expression in ‘HC’ and ‘QG’ mature fruits; Figure S11. Expression and methylation levels of genes involved in soluble sugar and organic acid metabolism; Table S1. Primer sequences used in this study; Table S2. Details of the bisulfite sequencing data for the ‘HC’ and ‘QG’ mature apple fruits; Table S3. Genome coverage in ‘HC’ and ‘QG’ mature fruits; Table S4. Details of RNA sequencing data for the ‘MC’ and ‘QG’ mature apple fruits; Table S5. Expression level of soluble sugar and organic acid related genes between ‘HC’ and ‘QG’ mature fruits.
