# Supplementary material for: Combined Profiling of Transcriptome and DNA Methylome Reveal Genes Involved in Accumulation of Soluble Sugars and Organic Acid in Apple Fruits
Source: Foods. 2021 Sep 16;10(9):2198. doi: 10.3390/foods10092198 (PMC8467953; doi:10.3390/foods10092198)
Supplement: Supplementary file 1 [file foods-10-02198-s001.zip › Supplimental figures.pdf]

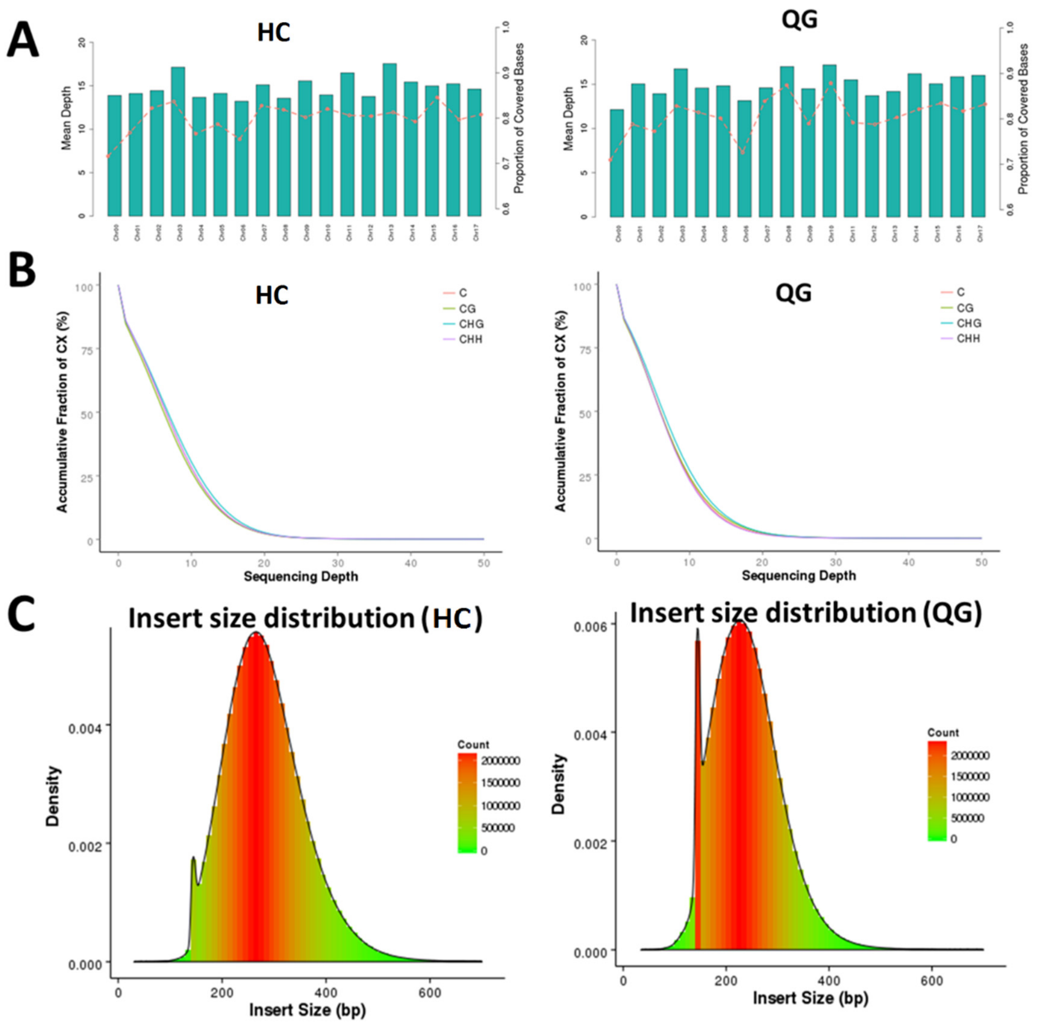

Figure S1. Characterization of bisulfite sequencing data in 'HC' and 'QG' mature fruits. A: Bisulfite sequencing data for the mean depth and proportion of covered bases in each chromosome for 'HC' and 'QG' mature fruits. B: Accumulative fraction of CX (%) based on the sequencing depth for 'HC' and 'QG' mature fruits. C: Insert size distribution for the bisulfite sequencing data for 'HC' and 'QG' mature fruits.

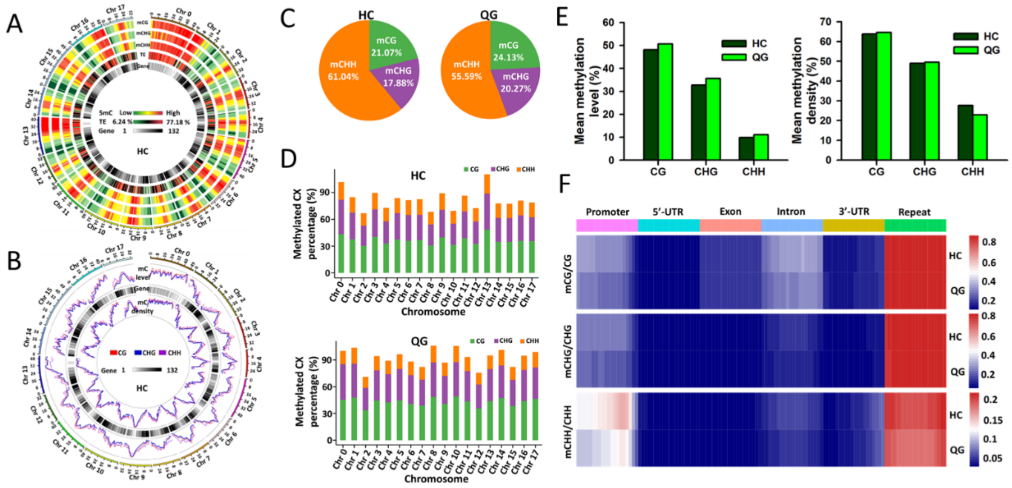

Figure S2. Details regarding the ‘HC’ and ‘QG’ mature fruit epigenomes. A: Circos plots of the methylation density for each chromosome. Track order: density plot of 5mC in the CG, CHG, and CHH contexts; TE density; and gene density for each chromosome in ‘HC’ mature fruits. B: Density of 5mC in the CG, CHG, and CHH contexts in the gene body regions of each chromosome in ‘HC’ mature fruits. C: Relative proportions of mC in the CG, CHG, and CHH contexts in ‘HC’ and ‘QG’ mature fruits. D: Methylated mC rate in the CG, CHG, and CHH contexts in ‘HC’ and ‘QG’ mature fruits. E: Mean methylation level and density in the CG, CHG, and CHH contexts in ‘HC’ and ‘QG’ mature fruits. F: Heat maps of DNA methylation levels in promoter, 5’- or 3’-UTR, exon, intron, and repeat regions in ‘HC’ and ‘QG’ mature fruits.

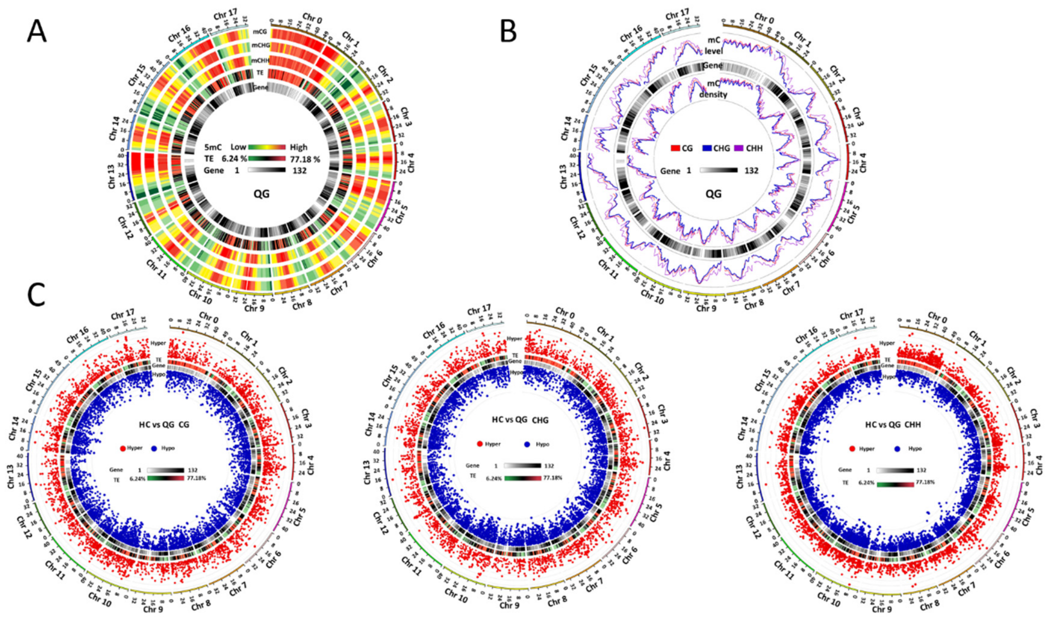

Figure S3. Detail information of epigenome in ‘HC’ and ‘QG’ mature fruits. A: Circos plots of methylation density for each chromosome. Track order: density plot of 5mC in CG, CHG and CHH contexts; the density of TEs, and gene density of each chromosome in ‘QG’ mature fruits. B: Density of 5mC in the CG, CHG and CHH contexts in the gene-body region on each chromosome in ‘QG’ mature fruits. C: Circos plot showed the hyper-and hypo-DMRs of CG, CHG and CHH contexts between ‘HC’ and ‘QG’ mature fruits.

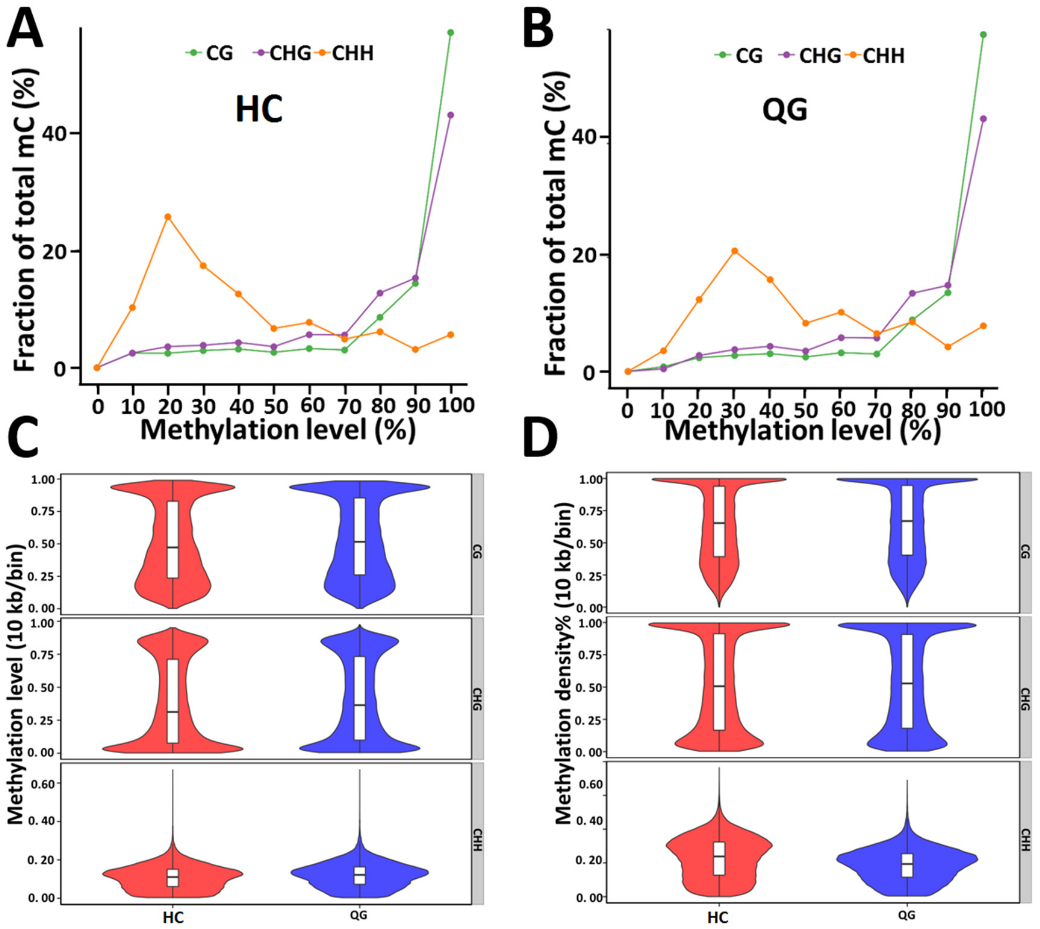

Figure S4. Methylation patterns of CG, CHG and CHH contexts in ‘HC’ and ‘QG’ mature fruits. A: Distribution of methylation levels in the CG, CHG, and CHH contexts in ‘HC’ and ‘QG’ mature fruits. B: Methylation levels and densities in the CG, CHG, and CHH contexts in ‘HC’ and ‘QG’ mature fruits.

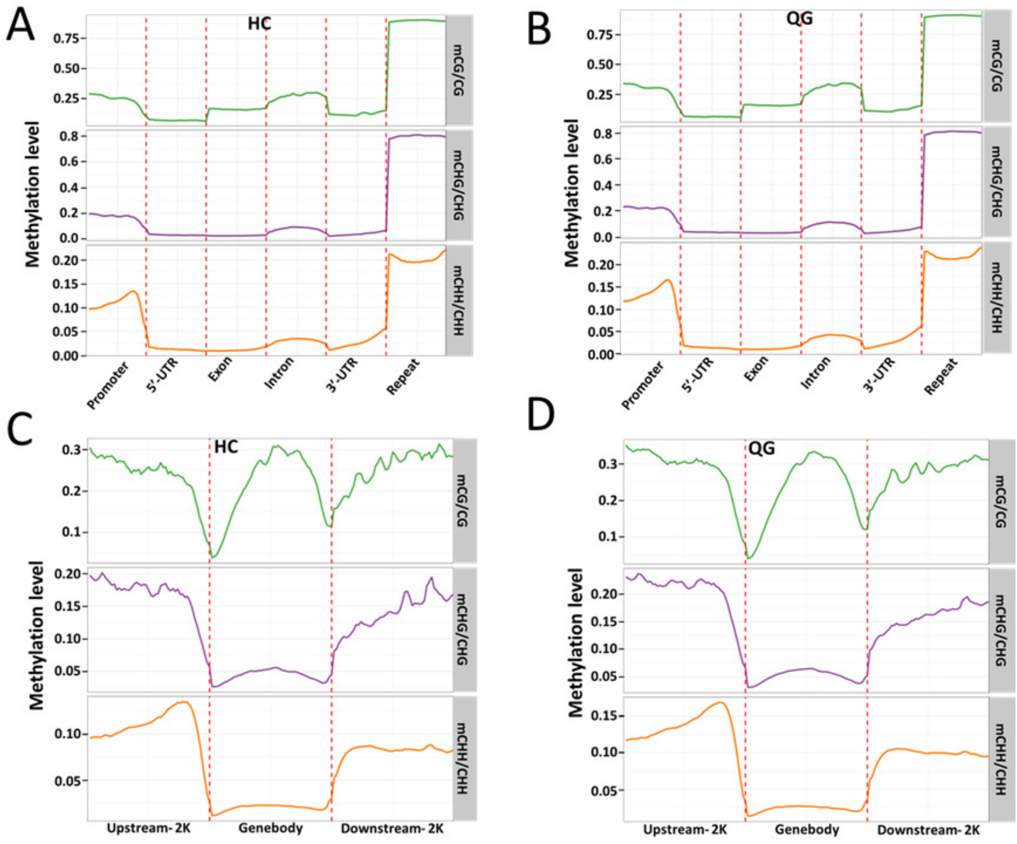

Figure S5. DNA methylation pattern in different genomic regions in 'HC' and 'QG' mature fruits. A, B: Distribution of DNA methylation levels among promoters, exons, introns, and repeat regions. C, D: Methylation levels (%) among gene-bodies and their 2-kb upstream and downstream regions in 'HC' and 'QG' mature fruits.

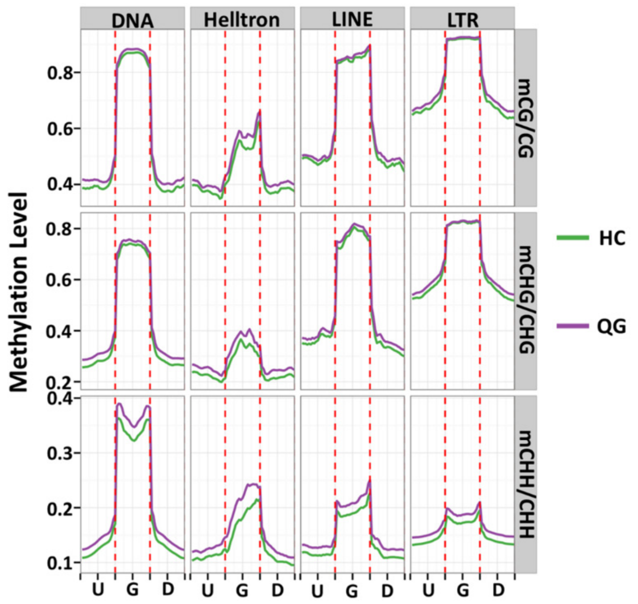

Figure S6. Methylation patterns of TEs in 'HC' and 'QG' mature fruits. U and D: 2 kb upstream and downstream regions, respectively; G: gene body regions.

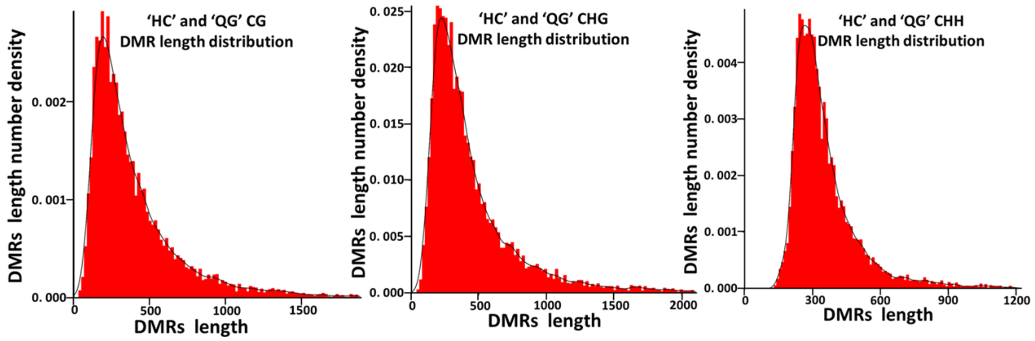

Figure S7. Distribution of DMR lengths in the CG, CHG, and CHH contexts in 'HC' and 'QG' mature fruits.

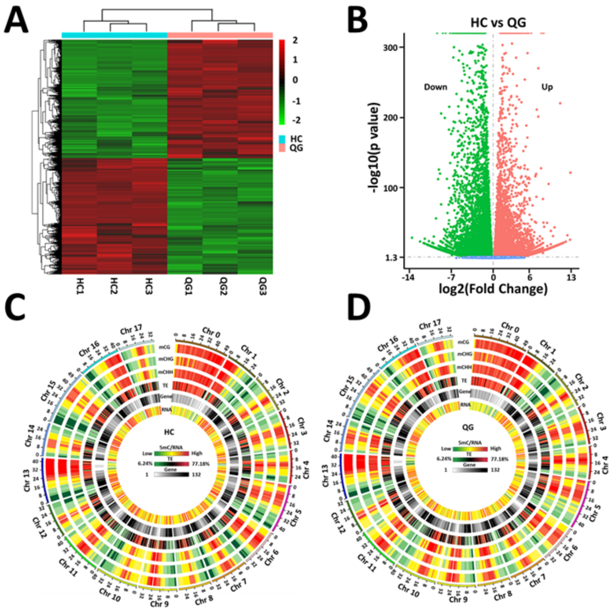

Figure S8. Differentially expressed genes (DEGs) between ‘HC’ and ‘QG’ mature fruits. A: Heatmap cluster of DEGs between ‘HC’ and ‘QG’ mature fruits. B: Volcano plot of DEGs between ‘HC’ and ‘QG’ mature fruits. C and D: Circos plots of DNA methylation and gene density of each chromosome. Track order: methylation density of CG, CHG and CHH contexts in ‘HC’ and ‘QG’ mature fruits, respectively.

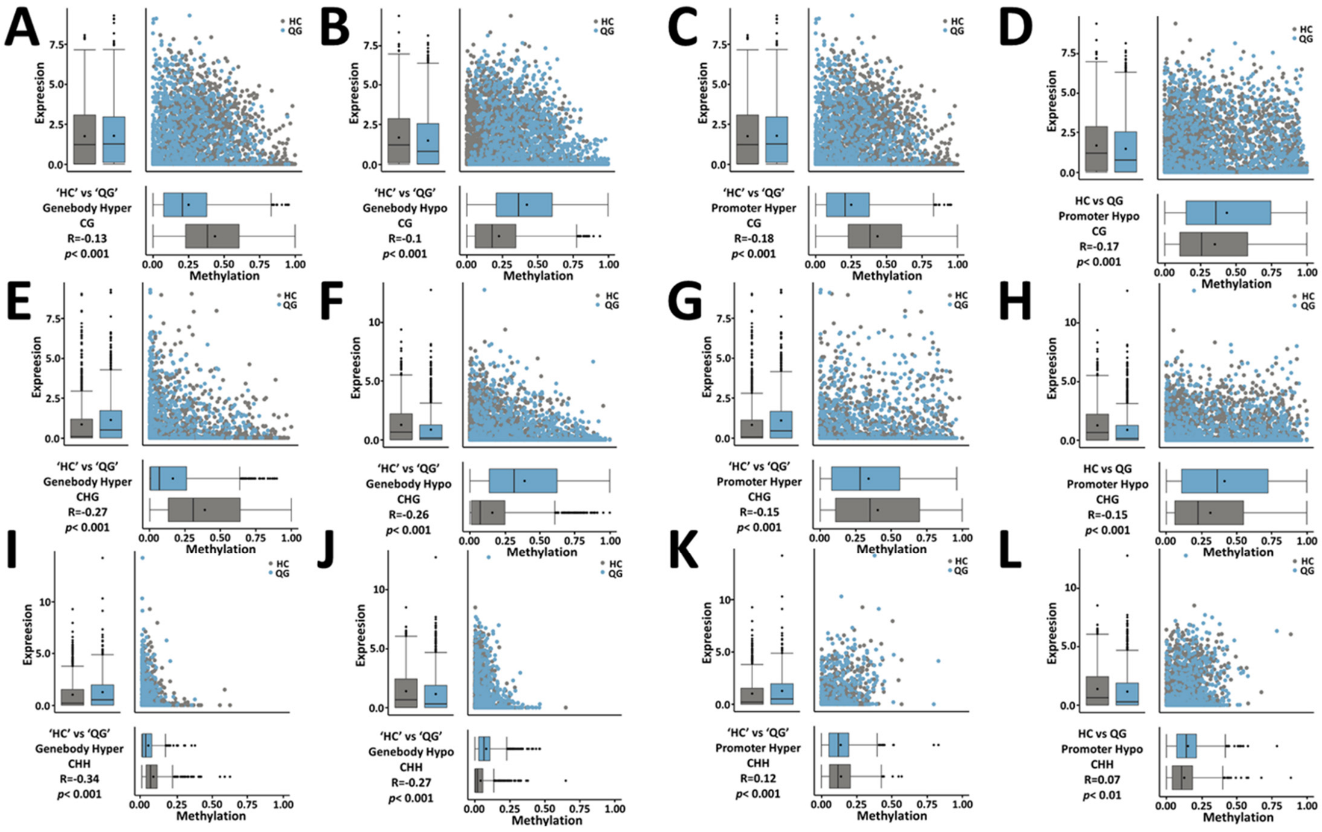

Figure S9. Association analysis between DNA methylation levels and gene expression of CH, CHG and CHH contexts in DMR gene bodies and DMR promoters between 'HC' and 'QG' mature fruits.

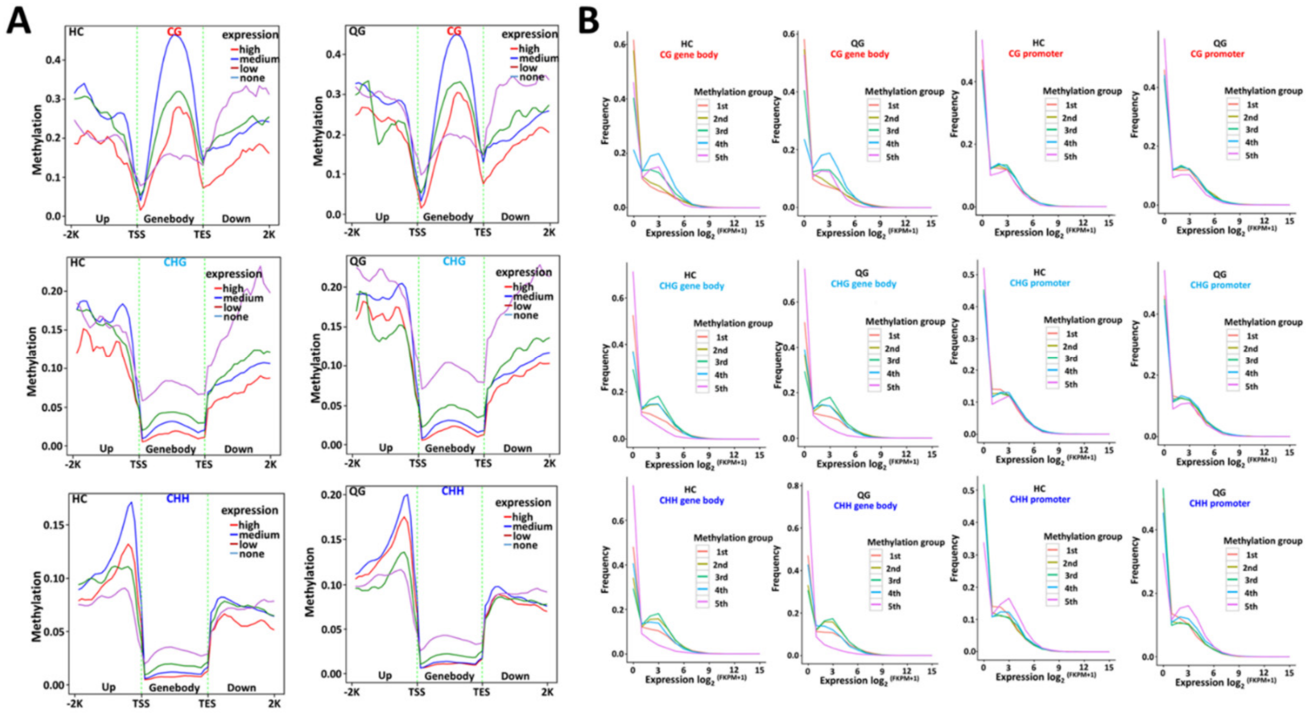

Figure S10. Correlation between DNA methylation and gene expression in 'HC' and 'QG' mature fruits. A: Distribution of methylation levels in the CG, CHG, and CHH contexts in different genomic regions for the following gene expression levels: high expression (FPKM ≥ 75% FPKM), medium expression (25% FPKM ≤ FPKM < 75% FPKM), low expression (1 ≤ FPKM < 25% FPKM), and no expression (none; FPKM < 1). B: Expression profiles of methylated genes in 'HC' and 'QG' mature fruits. The methylated genes were divided into the following five groups based on the methylation levels in promoter and gene body regions: group 1 (methylation level < 20%), group 2 (20% ≤ methylation level < 40%), group 3 (40% ≤ methylation level < 60%), group 4 (60% ≤ methylation level < 80%), and group 5 (methylation level ≥ 80%).

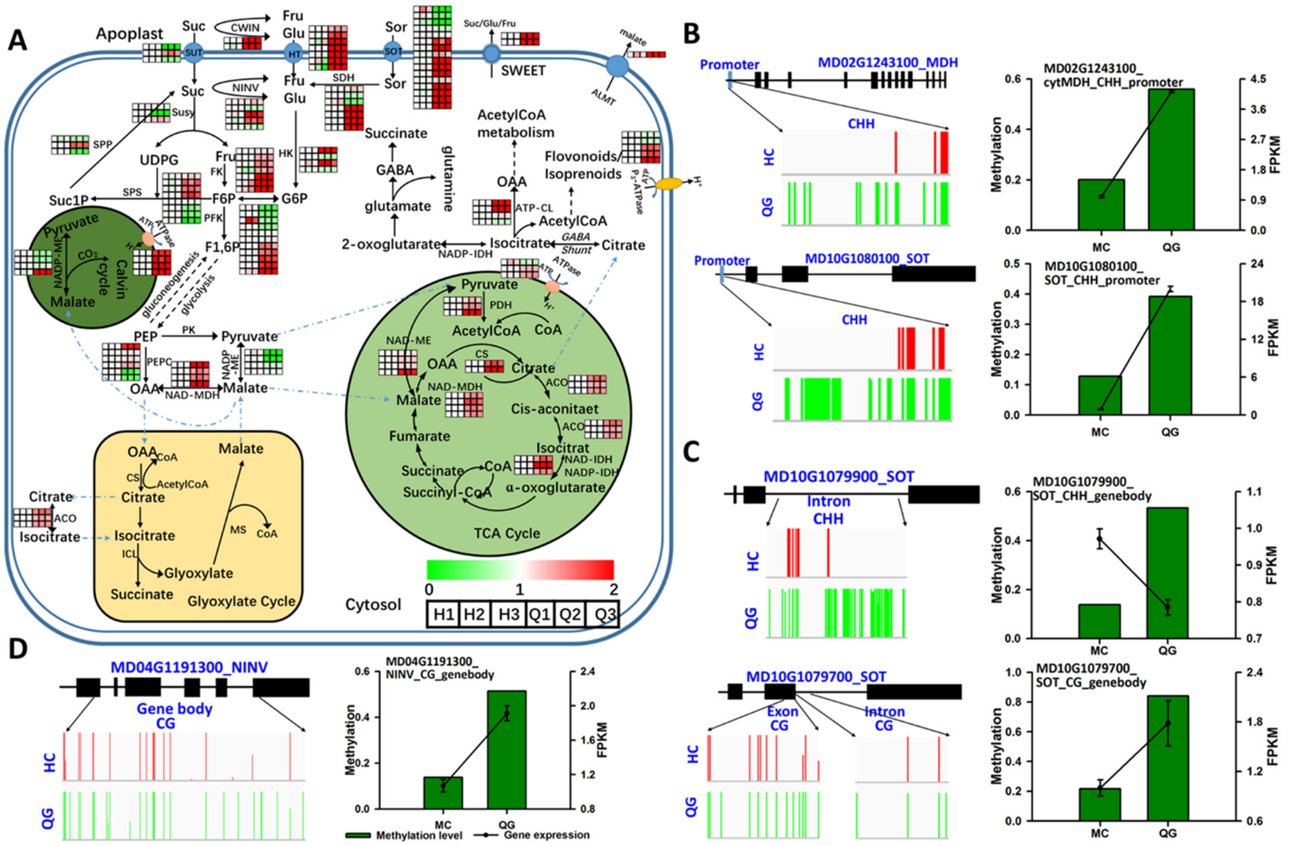

Figure S11. Expression and methylation levels of genes involved in soluble sugar and organic acid metabolism. A: Differentially expressed genes involved in soluble sugar and organic acid metabolism between ‘HC’ and ‘QG’ mature fruits. B: Integrative genomics viewer (IGV) snapshots of DNA methylation and gene expression in ‘HC’ and ‘QG’ mature fruits.
